# Smartphone-Based Psychotherapeutic Micro-Interventions to Improve Mood in a Real-World Setting

**DOI:** 10.3389/fpsyg.2016.01112

**Published:** 2016-07-28

**Authors:** Gunther Meinlschmidt, Jong-Hwan Lee, Esther Stalujanis, Angelo Belardi, Minkyung Oh, Eun Kyung Jung, Hyun-Chul Kim, Janine Alfano, Seung-Schik Yoo, Marion Tegethoff

**Affiliations:** ^1^Division of Clinical Psychology and Epidemiology, Department of Psychology, University of BaselBasel, Switzerland; ^2^Faculty of Medicine, Ruhr-University BochumBochum, Germany; ^3^Department of Brain and Cognitive Engineering, Korea UniversitySeoul, South Korea; ^4^Division of Clinical Psychology and Psychiatry, Department of Psychology, University of BaselBasel, Switzerland; ^5^Department of Radiology, Brigham and Women's Hospital, Harvard Medical SchoolBoston, MA, USA; ^6^Incheon St. Mary's Hospital, The Catholic University of KoreaIncheon, South Korea

**Keywords:** behavioral intervention technology, ehealth, health information technology, information and communication technology, Internet- and mobile-based intervention, mental disorder, mhealth, wireless health

## Abstract

**Background:** Using mobile communication technology as new personalized approach to treat mental disorders or to more generally improve quality of life is highly promising. Knowledge about intervention components that target key psychopathological processes in terms of transdiagnostic psychotherapy approaches is urgently needed. We explored the use of smartphone-based micro-interventions based on psychotherapeutic techniques, guided by short video-clips, to elicit mood changes.

**Method:** As part of a larger neurofeedback study, all subjects—after being randomly assigned to an experimental or control neurofeedback condition—underwent daily smartphone-based micro-interventions for 13 consecutive days. They were free to choose out of provided techniques, including viscerosensory attention, emotional imagery, facial expression, and contemplative repetition. Changes in mood were assessed in real world using the Multidimensional Mood State Questionnaire (scales: good–bad, GB; awake–tired, AT; and calm–nervous, CN).

**Results:** Twenty-seven men participated on at least 11 days and were thus included in the analyses. Altogether, they underwent 335, generally well-tolerated, micro-intervention sessions, with viscerosensory attention (178 sessions, 53.13%) and contemplative repetition (68 sessions, 20.30%) being the most frequently applied techniques. Mixed models indicated that subjects showed better mood [GB: *b* = 0.464, 95%confidence interval (CI) [0.068, 0.860], *t*_(613.3)_ = 2.298, *p* = 0.022] and became more awake [AT: *b* = 0.514, 95%CI [0.103, 0.925], *t*_(612.4)_ = 2.456, *p* = 0.014] and calmer [CN: *b* = 0.685, 95%CI [0.360, 1.010], *t*_(612.3)_ = 4.137, *p* < 0.001] from pre- to post-micro-intervention. These mood improvements from pre- to post-micro-intervention were associated with changes in mood from the 1st day until the last day with regard to GB mood (*r* = 0.614, 95%CI [0.297, 0.809], *p* < 0.001), but not AT mood (*r* = 0.279, 95%CI [−0.122, 0.602], *p* = 0.167) and CN mood (*r* = 0.277, 95%CI [0.124, 0.601], *p* = 0.170).

**Discussion:** Our findings provide evidence for the applicability of smartphone-based micro-interventions eliciting short-term mood changes, based on techniques used in psychotherapeutic approaches, such as mindfulness-based psychotherapy, transcendental meditation, and other contemplative therapies. The results encourage exploring these techniques' capability to improve mood in randomized controlled studies and patients. Smartphone-based micro-interventions are promising to modify mood in real-world settings, complementing other psychotherapeutic interventions, in line with the precision medicine approach. The here presented data were collected within a randomized trial, registered at ClinicalTrials.gov (Identifier: NCT01921088) https://clinicaltrials.gov/ct2/show/NCT01921088.

## Introduction

Mental disorders are one of the leading global causes of disability (Murray et al., 2012). Besides the personal suffering, their direct and indirect economic costs are tremendous (Wittchen et al., 2011; Olesen et al., 2012). A prominent consortium of researchers, advocates, and clinicians identified key “grand challenges in global mental health” in terms of major research priorities for improving the lives of people with mental illnesses around the world (Collins et al., 2011). Notably, one of the prioritized goals is to improve treatments and expand access to mental health care, with the development of mobile and Internet technologies to increase access to evidence-based care being among the top challenges (Collins et al., 2011). This need is underscored by the fact that in countries, regardless of their economic status, the demand for individual face-to-face psychotherapy is already exceeding or will exceed mental health service supply in the future (Kazdin and Blase, 2011). Therefore, new forms of treatment are required that can complement or expand our current approaches in treating people who suffer from mental disorders (Kazdin and Blase, 2011; Kostkova, 2015).

To this end, Internet-based psychotherapies have received considerable attention during the past decade, lowering the barrier to access mental health service. Most studies indicated that Internet-delivered interventions were efficacious in achieving positive behavioral change or symptom reduction, with no clear evidence of superiority or inferiority as compared to face-to-face interventions (Cuijpers et al., 2010; Griffiths et al., 2010; Richards and Richardson, 2012; Andersson et al., 2014; Riper et al., 2014; Ebert et al., 2015; Richards et al., 2015; Kuester et al., 2016; Melioli et al., 2016; Olthuis et al., 2016; Zachariae et al., 2016).

The advent of mobile information technologies has taken this low-barrier approach to the next level. In the year 2020, 70% of the world's population will use a smartphone (Ericsson, 2015). The core features of smartphones and other mobile devices are that they are running most of the time, are used in a variety of situations during daily life, and ensure a broad reachability of their users beyond calls, e-mails, short messaging, or instant messaging. Unlike the dissemination of many other technologies, the rapid uptake of mobile phones has not been restricted to developed countries (Kay et al., 2011). Furthermore, mobile phones are the preferred means of communication among young people, the age group most unlikely to seek treatment (Oliver et al., 2005). However, some target populations, such as veterans, that experience mental health service gaps may also be more difficult to reach via smartphone-based interventions, as compared to the general population (Klee et al., 2016). Smartphones are increasingly complex, computationally powerful, sensory-rich, and integrated with social networking (Morris and Aguilera, 2012). These factors make them ideal for the delivery of mental health information, digital psychotherapeutic techniques and support anywhere, in real-time and when needed, the latter identified amongst others using sensors integrated in the smartphone (McClernon and Roy Choudhury, 2013). This is in line with the “precision medicine approach,” aiming to provide the right treatment, at the right time, and for the right person (Insel, 2014; Collins and Varmus, 2015). Integrating smartphones in mental healthcare provides a wealth of opportunities, including to overcome the innovation gap by allowing for “disruptive innovation” (Bower and Christensen, 1995), and to provide the basis for new, personalized forms of treatment (Ehrenreich et al., 2011; Zeevi et al., 2015).

Modifying mood or inducing certain mood states in the laboratory, using different approaches in non-clinical samples, has a long-standing history in psychological research (Velten, 1968; Martin, 1990; Schaefer et al., 2010). However, there are only few studies that examined the use of exclusively smartphone-based interventions to modify mood or affective states in healthy populations (e.g., Cipresso et al., 2012); which is in contrast to the large number of studies using smartphones for mood assessment (e.g., Asselbergs et al., 2016). However, a better understanding on how smartphones may be used to modify mood in healthy subjects may provide an important basis for its future application in clinical samples.

Notably, even though an increasing number of mobile applications (apps) that claim to target mental health are available in software repositories (Mani et al., 2015; Nicholas et al., 2015; Shen et al., 2015), as yet, studies that evaluate the effects of applying smartphones as a means of behavior modification are relatively scarce (Donker et al., 2013; Mohr et al., 2013a; Harrison and Goozee, 2014; Mani et al., 2015; Olff, 2015; Torous and Powell, 2015; Bakker et al., 2016). Initial studies provide evidence that smartphone-based interventions have the potential to reduce symptoms of mental disorders, such as anxiety, depression, schizophrenia, and substance use disorders (Watts et al., 2013; Ben-Zeev et al., 2014; Gustafson et al., 2014; Ly et al., 2014; Ahmedani et al., 2015). Further, there is first evidence that mobile technology, including smartphone-based applications, can boost the efficacy of psychotherapy and behavioral interventions (Lindhiem et al., 2015). In sum, further research on smartphone-based interventions in non-clinical samples is highly warranted, and may provide an important basis for future studies and applications, aiming at improving and facilitating prevention and treatment of mental disorders, which has the potential to complement established treatment approaches, serving great clinical and societal relevance.

One particular challenge in the field of mobile mental health research is the mismatch of the paces of research and technology development, with rather long timeframes of classical randomized controlled trials (RCTs), the gold standard of research designs to determine the efficacy of an intervention, with a median duration of more than 5 years from initial enrollment to publication and much longer timeframes until implementation into routine care (Ioannidis, 1998; Riley et al., 2013; Clough and Casey, 2015). This has led to the call for new frameworks and refinement of mobile mental health research (Kumar et al., 2013; Riley et al., 2013; Mohr et al., 2013a,b; Ben-Zeev et al., 2015; Clough and Casey, 2015; Nicholas et al., 2015; Bakker et al., 2016). Classical RCTs evaluate a well-circumscribed intervention; hence modifications of the intervention require conducting a new RCT. One solution to this problem, we believe, is to evaluate core psychotherapeutic components and key features of interventions, which can then guide the assembly of the intervention as a whole, if desired still followed by an RCT. To this end, studies that focus on the evaluation of important elements, characteristics, and principles of smartphone-based interventions, starting with non-clinical samples and later being applied to patients, may be of great importance (Mohr et al., 2014; Alkhaldi et al., 2016; Bakker et al., 2016).

The idea to focus on core intervention components is in line with transdiagnostic treatment approaches, which center on core disease mechanisms to improve the understanding and treatment of mental disorders (Wilamowska et al., 2010; Thompson-Hollands et al., 2014; Newby et al., 2015, 2016). One central target of psychotherapeutic interventions is the improvement of mood, with mood disturbances being the key symptom of a variety of mental disorders (American Psychiatric Association, 2013). Furthermore, mood plays a key role in the quality of daily life, and influences personal and social adjustment and physical health, social interactions, and problem solving (Fredrickson, 2004; Shallcross et al., 2010). Hence, the advancement of easily applicable interventions to improve mood is of paramount importance.

Our goal was to explore in a real-world setting, in a non-clinical sample, the use of smartphone-based micro-interventions and related changes in mood. We thereby applied micro-interventions in form of psychotherapeutic techniques that have already been used as components of face-to-face psychotherapy (see Paredes et al., 2014), guided by short video-clips of <5 min duration. More specifically, we aimed at estimating changes in mood and hypothesized that mood would improve from pre- to post-micro-intervention. Furthermore, we evaluated whether these changes were related to changes in mood from the first to the last micro-intervention day, and finally, whether they varied over time and between techniques. The analyzed data were collected from 13 daily micro-intervention sessions, as part of a larger neurofeedback study, in which two real-time functional magnetic resonance imaging neurofeedback (RT-fMRI NF) sessions were conducted, one before all daily micro-intervention sessions and one after, separated by 14 days.

## Materials and methods

### Outline of the study procedure

#### Overall study procedure

The data presented here were collected within a randomized trial, registered at ClinicalTrials.gov (Identifier: NCT01921088) https://clinicaltrials.gov/ct2/show/NCT01921088. The aim of this larger study was to assess the application of real-time functional magnetic resonance imaging neurofeedback (RT-fMRI NF) to modulate the response to an acute stressor in form of the Stroop color word interference task. RT-fMRI NF is a type of self-regulation technique that provides an individual with feedback about specific brain activity using functional magnetic resonance imaging in connection with a related behavior; The underlying assumption at the core of this practice is that through RT-fMRI NF a subject can learn to regulate neural activity and related mental functions (see Thibault et al., 2016).

The institutional review board of Korea University approved the study protocol. All subjects gave written informed consent in accordance with the Declaration of Helsinki. The study was conducted between August and October 2013 at the facilities of Korea University, Seoul, Republic of Korea [Resource Identifier (RRID): SCR_004095].

**Figure 1:**
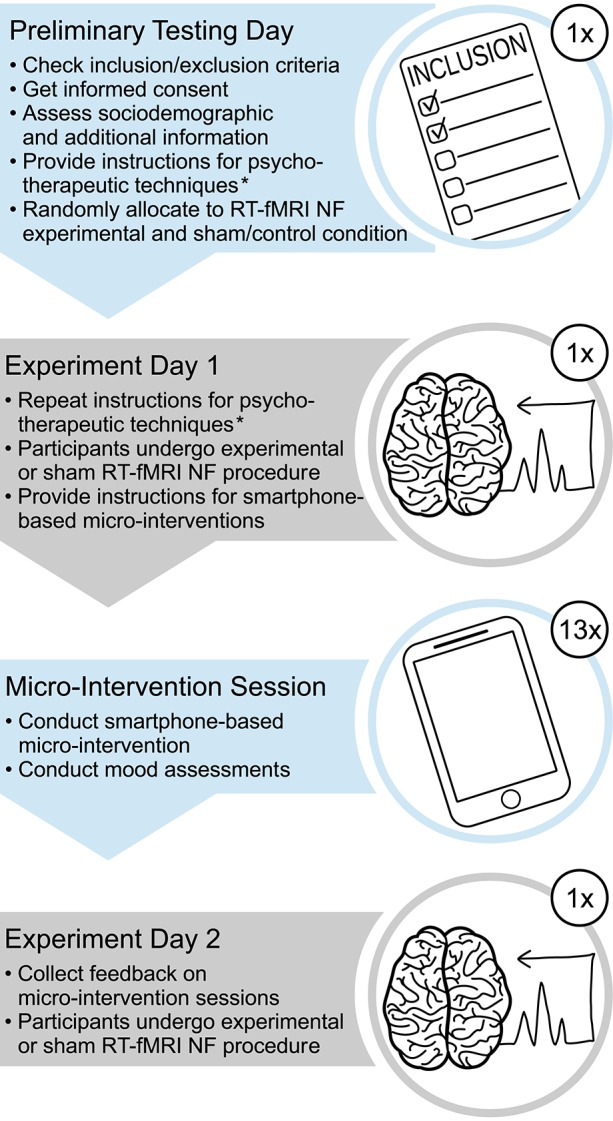
**Outline of the larger study**. The data presented here were collected during the preliminary testing day and the smartphone-based micro-interventions, with the exception of the feedback on the micro-interventions that was collected before the RT-fMRI NF procedure on experiment day 2. ^*^We instructed participants in four psychotherapeutic techniques, first at the preliminary testing day, then at the beginning of the session at experiment day 1, and briefly reiterated these instructions at the end of this session; RT-fMRI NF, real-time functional magnetic resonance imaging neurofeedback.

The whole study consisted of three laboratory visits and 13 days of ambulatory smartphone-based micro-interventions using psychotherapeutic strategies, the latter following the second laboratory visit on which the RT-fMRI NF procedure was applied for the first time (see Figure 1; for a brief overview of the whole study, please refer to Supplementary Material Data Sheet 1). The data presented here were collected during the preliminary testing day and the smartphone-based micro-interventions, with the exception of the feedback on the micro-interventions that was collected before the RT-fMRI NF procedure on experiment day 2. First, we screened subjects interested in study participation during a telephone interview for any history of neurological or mental disorders and invited those eligible to a laboratory visit, the preliminary testing day, on which we verified whether subjects met all eligibility criteria (see below). Subjects fulfilling eligibility criteria and interested in study participation were asked to provide additional data via questionnaires, were instructed in psychotherapeutic techniques (see section below) to be practiced in different phases of the study, and invited to two further laboratory visits (14 days apart from each other) for a RT-fMRI NF experiment. Between these two experiment days, subjects participated in smartphone-based micro-interventions, during which they practiced the psychotherapeutic techniques they had previously learned on the preliminary testing day and experiment day 1, respectively.

#### Preliminary testing day

At the preliminary testing day, we first outlined the whole study procedure to the subjects and collected their written informed consent. Then, we had them practice four psychotherapeutic techniques (for details, see below), which they later applied during the RT-fMRI NF experiment and the micro-interventions. In this first introduction to the techniques, we used detailed instructions and handed out copies with the written instructions to the participants, so that they could follow the text while we explained the techniques. Next, we explained them all other tasks relevant for the RT-fMRI NF experiment procedure (details available from the authors on request). We then asked the participants to fill in a set of questionnaires and checklists to verify their eligibility to the experiment and gather additional information (e.g., sociodemographic data). A detailed description of those questionnaires relevant for this publication is given below. The experimenter then looked through the results and decided upon inclusion of participants. In case of inclusion, the experimenter and subject made an appointment for the next visit at the laboratory for experiment day 1 (6 weeks later at maximum).

#### Psychotherapeutic techniques

We instructed the participants in four psychotherapeutic techniques, first at the preliminary testing day, then at the beginning of the session at experiment day 1, and briefly reiterated these instructions at the end of this session. We told the participants that they might find these techniques useful to accomplish the upcoming tasks during the RT-fMRI NF experiment in terms of modulating their brain activity as well as their stress level. The following four techniques were instructed: (i) viscerosensory attention, (ii) emotional imagery, (iii) facial expression, and (iv) contemplative repetition. Additionally, participants were allowed to use (v) any other individual technique that they felt would be helpful. A brief outline of the techniques, as provided at the end of experiment day 1, is depicted in Table 1. In brief, (i) viscerosensory attention consisted of shifting attention toward vs. away from bodily sensations, for example heartbeat or breathing; (ii) emotional imagery consisted of imagining emotionally positive (e.g., great holidays, a beloved person), negative (e.g., a stressful exam, a conflict) or neutral (e.g., a bus ride, reading the newspaper) situations; (iii) facial expression consisted of making different emotional facial expressions, e.g., a happy, angry, or neutral face; and (iv) contemplative repetition consisted of repeating a short simple sentence or word over and over again, or slowly and repeatedly counting from 1 to 10. The shifting between different extremities, as instructed for viscerosensory attention, emotional imagery, and facial expression, was to exploit a preferably large scope of modifiability. To ensure that subjects well remembered the techniques for application during the smartphone-based micro-interventions, at the end of experiment day 1, we asked subjects (i) to take some time to vividly remember the technique that they had just applied in the scanner and that worked best for them, and to briefly describe this technique in written form; (ii) to think of and write down a keyword that might help them to call up this technique once they would apply it during the subsequent micro-intervention sessions; and (iii) to think of a picture that might help them to recall this technique during the micro-intervention sessions, and to describe it in words or draft it. All four psychotherapeutic techniques have been shown to be related to changes in mood (Kleinke et al., 1998; Holmes et al., 2006; Lane et al., 2007; Pollatos et al., 2015), with potential for the treatment of mental disorders (Ito et al., 2001; Holmes et al., 2007; Orme-Johnson and Barnes, 2014; Lin et al., 2015).

**Table 1:** **Description of techniques applied during the micro-interventions**.

**Technique**	**Instruction**
Viscerosensory attention	“*Shift your attention toward vs. away from bodily sensations, for example your heartbeat, breathing, or feelings in stomach. Keep your attention focused on each sensation for a while.”*
Emotional imagery	“*Imagine emotionally positive, negative, or neutral situations, and shift your attention between them. For example think of a beloved person, a stressful exam or a conflict, or a bus ride. Keep your attention focused on each situation for a while.”*
Facial expression	“*Make different emotional expressions with your face and keep each for a while (e.g., happy face, angry face, neutral face).”*
Contemplative repetition	“*Repeat a short, very easy sentence, or slowly count from 1 to 10 (repeat this over and over again).”*
Other technique	“*Remember the strategy that you have successfully practiced in the scanner. Please concentrate and practice this strategy during the next minutes.”*

#### Smartphone-based micro-interventions

To familiarize the participants with the smartphone-based micro-intervention, we asked all subjects to undergo one micro-intervention session for training purposes, while still in the laboratory at the end of experiment day 1. Data collected during this training session were not included in our analyses.

On the 13 days between experiment day 1 and 2, each participant underwent one session of smartphone-based micro-intervention per day during their daily life, in which he applied one of the psychotherapeutic techniques outlined above. We instructed subjects to use their own smartphones for participating in the micro-intervention sessions (see Supplementary Material Table 1 for additional information on smartphone types, operating systems, and Internet browsers used). Subjects were free to choose the time of day at which they underwent the micro-intervention session. The time window during which the subjects had to undergo the daily micro-intervention session started each day at 0800 h when they received the invitation-e-mail including the personalized and day-specific hyperlink for access to the micro-intervention session. This hyperlink expired at 0300 h on the following day. In addition to the daily invitation-e-mail at 0800 h, subjects received a reminder-e-mail at 2000 h if they had not yet participated since the last invitation.

We used EFS Survey 10.0 (Questback GmbH, Berlin, Germany) to conduct the smartphone-based micro-interventions, including instructions, presentation of a video-clip, and collection of questionnaire data, as well as for automatically sending the invitation- and reminder-e-mails.

The detailed procedure of each session was as follows: (1) Subjects used their smartphones to connect via internet browser, using a personalized hyperlink provided in the daily invitation or reminder e-mails, to the server hosted by Questback. (2) We instructed the subjects by text display to seek a quiet place allowing them to concentrate on the micro-intervention, and to ensure having a stable Internet connection. Furthermore, we instructed them that the end of the micro-intervention would be signalized by a sound, and that they should therefore ensure to plug in their headphones or set the loudspeakers of their smartphone on high volume, if possible, and that alternatively, the end of the micro-intervention would also be recognizable by visual cues. (3) We asked the subjects to enter their individual subject ID that we had previously provided, as well as a self-generated personal code that they had already generated during the preliminary testing day. This code allowed verifying subject identity. (4) Subjects responded to the Multidimensional Mood State Questionnaire (MDMQ), described in more detail below, and the self-assessment manikin (SAM) scales (Bradley and Lang, 1994). (5) We instructed the subjects to prepare for the micro-intervention, including (i) asking them to remember the technique that they successfully applied during experiment day 1 and telling them that they should use this technique on each of the daily micro-intervention sessions, (ii) instructing them that a micro-intervention session would consist of two rounds lasting 2 min each, interrupted by a pause of 30 s and that in order to start with the session, they should click on the “play”-button of the video player; (iii) asking them—if their Internet connection was weak—to click on the “stop”-button to wait until the player had completely loaded the video, then to reset the video, and to the start the video by clicking on “play” again; and (iv) informing them that the end of the micro-intervention session was signalized by a sound and visually announced in the video, and instructing them not to click on “Continue” before they heard the sound or before the end of the video was reached, as this is important to ensure a standardized duration of the session for each participant and on each day; (v) After this, we asked subjects to select the psychotherapeutic technique they wanted to use during this session (for details, see previous sections). (6) Then, subjects underwent the micro-intervention by following the instructions provided within a short video-clip (duration each: ~4 min 40 s), presented according to the technique that they wanted to apply (the video-clips are provided as Supplementary Material Video 1–5; details of the structure and content of the video-clips are as Supplementary Material Data Sheet 2; additional information regarding the video files as Supplementary Material Data Sheet 3). (7) Subjects again responded to the MDMQ and the SAM scales. (8) Then, subjects replied to two questions related to the micro-intervention session: first, they were asked how successful their session was today, with possible replies on a 5-level scale ranging from −2 (much less than expected/very bad) to +2 (much more than expected/very good). Second, they were asked how they could optimize their micro-intervention (e.g., conditions, motivation, timing, etc.), with an open answer format. The aim of this second question was to guide subjects toward individual optimization of their personal micro-intervention. (9) The session finished by thanking them for their participation in today's session and reminding them of the next micro-intervention session on the subsequent day (or of experiment day 2 on the last day of micro-intervention sessions).

For each page that EFS provided, it recorded a time-stamp, from which we were able to derive date and time of each micro-intervention session.

### Assessment instruments

#### Assessment of inclusion/exclusion criteria

We applied a set of well-established questionnaires (presented as paper-pencil questionnaires or electronically) to gather information from the participants along the study. Further, we used a set of short checklists to collect additional information, such as data regarding eligibility criteria and feedback regarding the smartphone-based micro-interventions.

To verify the eligibility criterion “right handedness” and the exclusion criterion “color-blindness,” we asked the study participants to fill in the Edinburgh Handedness Inventory (EHI; Oldfield, 1971) and the Ishihara test for color-blindness (Ishihara and Force, 1943), respectively, on the preliminary testing day.

#### Assessment of mood and feedback on micro-intervention sessions

We applied the 12-item MDMQ to assess current mood on three dimensions ranging from good to bad (GB), awake to tired (AT), and calm to nervous (CN). The MDMQ is the English version of the German *Mehrdimensionale Befindlichkeitsfragebogen* (MDBF; Steyer et al., 1997; Steyer, 2014), which is a well-established tool for the assessment of current mood, with very good psychometric properties, especially suited for repeated measures within short intervals. For each dimension, a score is calculated, ranging from 4 to 24. Depending on the dimension, high scores suggest positive affectivity, wakefulness, and calmness, respectively. We applied the MDMQ twice during each smartphone-based micro-intervention session, both before and after subjects practiced the psychotherapeutic technique.

We obtained feedback regarding the smartphone-based micro-intervention sessions at the beginning of experiment day 2, asking the subjects if they agreed with the four statements displayed in Supplementary Material Table 2. Additionally, participants were encouraged to provide further comments regarding the micro-intervention sessions.

Two researchers (AB and JA) independently entered all data from paper-pencil questionnaires into electronic spreadsheets, and a third researcher (ES) crosschecked their entries.

### Participants

We recruited participants from the student body of the Korea University. Advertisements for the study were posted on the university website and a local bulletin board. Participants had to fulfill the following eligibility criteria, which were based on the requirements of the larger RT-fMRI NF study: male, age 18–65 years, right-handed, no color-blindness, no history of cardiovascular or neurological diseases or mental disorders, sufficient English language skills to follow the experimental instructions, and self-reported at least minimal familiarity with smartphone-use to carry out the micro-interventions. The sample size was determined *a priori*, based on the requirements of the randomized trial assessing RT-fMRI NF effects, to provide sufficient statistical power to test the main hypotheses of the trial. Samples size estimates were based on previous studies, demonstrating large effect sizes within RT-fMRI NF paradigms (deCharms et al., 2005; Yoo et al., 2008; Kim et al., 2015). We estimated, by calculating *a priori* power analysis (using G^*^ Power 3, Faul et al., 2007, RRID: SCR_013726) that with *n* = 14 subjects in each condition, effects of *d* = 1.0 can be detected with sufficient power (1–ß > 0.80; given α = 0.05, one-sided test).

After completion of the study, each subject received 60,000 KRW (≈57 USD) in compensation for his participation. The compensation was split in three parts, for the participation at experiment day 1, smartphone-based micro-interventions, and experiment day 2, and paid out in part if the subject did not take part in the complete study.

### Statistical analyses

We checked the data for distribution properties and verified normality by inspecting histograms and qq-plots. For descriptive analyses, we calculated means and standard deviations for continuous normally distributed variables and absolute and relative frequencies for categorical variables with categories outlined in Table 2.

**Table 2:** **Characteristics of the study sample (***N*** = 27)**.

**Variable**	**Category**	***n***	(%)^*^
**CATEGORICAL VARIABLES**			
Marital status	Single	20	(74.07%)
	In a relationship	7	(25.93%)
Highest degree	High school or equivalent	24	(88.89%)
	Bachelor's degree	3	(11.11%)
Size of household (including participant)^**^	1	1	(3.85%)
	2	0	(0%)
	3	1	(3.85%)
	4	22	(84.62%)
	5	2	(7.69%)
“I am very experienced in using smartphones”	Strongly agree	7	(25.93%)
	Agree	14	(51.85%)
	Neutral	4	(14.81%)
	Disagree	1	(3.70%)
	Strongly disagree	1	(3.70%)
**Variable (unit)**	**Mean**	**(*SD*)**	**Range [min, max]**
**CONTINUOUS VARIABLES**			
Age (years)	24.32	(2.27)	[19.75, 28.70]
Full time education (years)	15.15	(1.38)	[12, 18]

* Percentages may not total 100 due to rounding;

** *Information from one subject missing; max, maximum; min, minimum; SD, standard deviation*.

As the values of the scales of the MDMQ were approximately normally distributed, transformation was not required. Twenty-six of the 27 participants applied the same psychotherapeutic technique across micro-intervention days, but one subject extensively varied the psychotherapeutic technique across days. Therefore, we did not enter psychotherapeutic technique as factor at the level of the micro-intervention day, but entered it at the participant level. To this end, we assigned each of the above-mentioned 26 participants to the psychotherapeutic technique category that they used, and created an additional category “mixed techniques” for the subject that extensively varied the psychotherapeutic techniques. As in four of the resulting six categories there were only few subjects (“emotional imagery”, *n* = 3; “facial expression”, *n* = 2; “other technique”, *n* = 2; and “mixed techniques”, *n* = 1), we collapsed these four categories, leading to the trichotomous variable “psychotherapeutic technique” with the three levels “viscerosensory attention” (*n* = 14), “contemplative repetition” (*n* = 5), and “other” (*n* = 8).

Each scale of the MDMQ was entered as outcome variable in separate linear mixed-effects models (Singer and Willett, 2003), to estimate mood changes from pre- to post-micro-intervention and across micro-intervention days, as well as differences in mood changes from pre- to post-micro-intervention across micro-intervention days and between psychotherapeutic techniques. Furthermore, we adjusted analyses for the condition (experimental or sham/control condition) to which subjects had been assigned within the larger randomized controlled trial from which the data was derived. Hence, we entered the following predictors into the model: (i) “pre- vs. post-micro-intervention”, (ii) “micro-intervention day” (dimensional, day 1–13), iii) “psychotherapeutic technique” (trichotomous, see above), and iv) “condition” (experimental vs. sham/control), as well as the interactions of “pre- vs. post micro-intervention” with “micro-intervention day”, “psychotherapeutic technique”, and “condition”. We entered random intercept and random slope parameters when this improved model fit, with the latter being assessed based on Akaike's Information Criterion (AIC; Singer and Willett, 2003). Furthermore, we tested whether entering a higher order polynomial of the variable “micro-intervention day” would improve model fit. We first fitted models including main and interaction effects, as outlined above. In case of the interaction effects being statistically not significant, we repeated analyses with main effects only, leading to the main results reported. We calculated 95% confidence intervals (CIs) using the Wald method. For the main mixed model analyses, we included all subjects that took part in at least 3 micro-intervention sessions. Mixed models accommodated further missing data.

To test whether average mood improvements from pre- to post-micro-interventions were associated with overall baseline mood improvements over all intervention days, we calculated Pearson product-moment correlation coefficients between the mean of the non-missing mood changes from pre- to post-micro-interventions averaged across days 2–12 and change in mood from pre-micro-intervention day 1 to pre-micro-intervention day 13, separately for GB, AT, and CN mood.

All tests were two-tailed and we set the significance level at 0.05. We used the statistical software package R (version 3.2.3 and above; R Project for Statistical Computing, RRID: SCR_001905; R Core Team, 2015) for all data analyses and statistical testing, including the packages to conduct the mixed models, “lme4” (Bates et al., 2014) and “optimx” (Nash and Varadhan, 2011), as well as further packages, required for data preparation and descriptive statistics “car” (Fox and Weisberg, 2011), “dplyr” (Wickham and Francois, 2015), “haven” (Wickham and Miller, 2015), “Hmisc” (Frank and Dupont, 2015), “lmerTest” (Kuznetsova et al., 2015), “lsmeans” (Lenth, 2016), “pastecs” (Grosjean and Ibanez, 2014), and “tidyr” (Wickham, 2015).

## Results

### Flow and descriptive information on study participants

**Figure 2:**
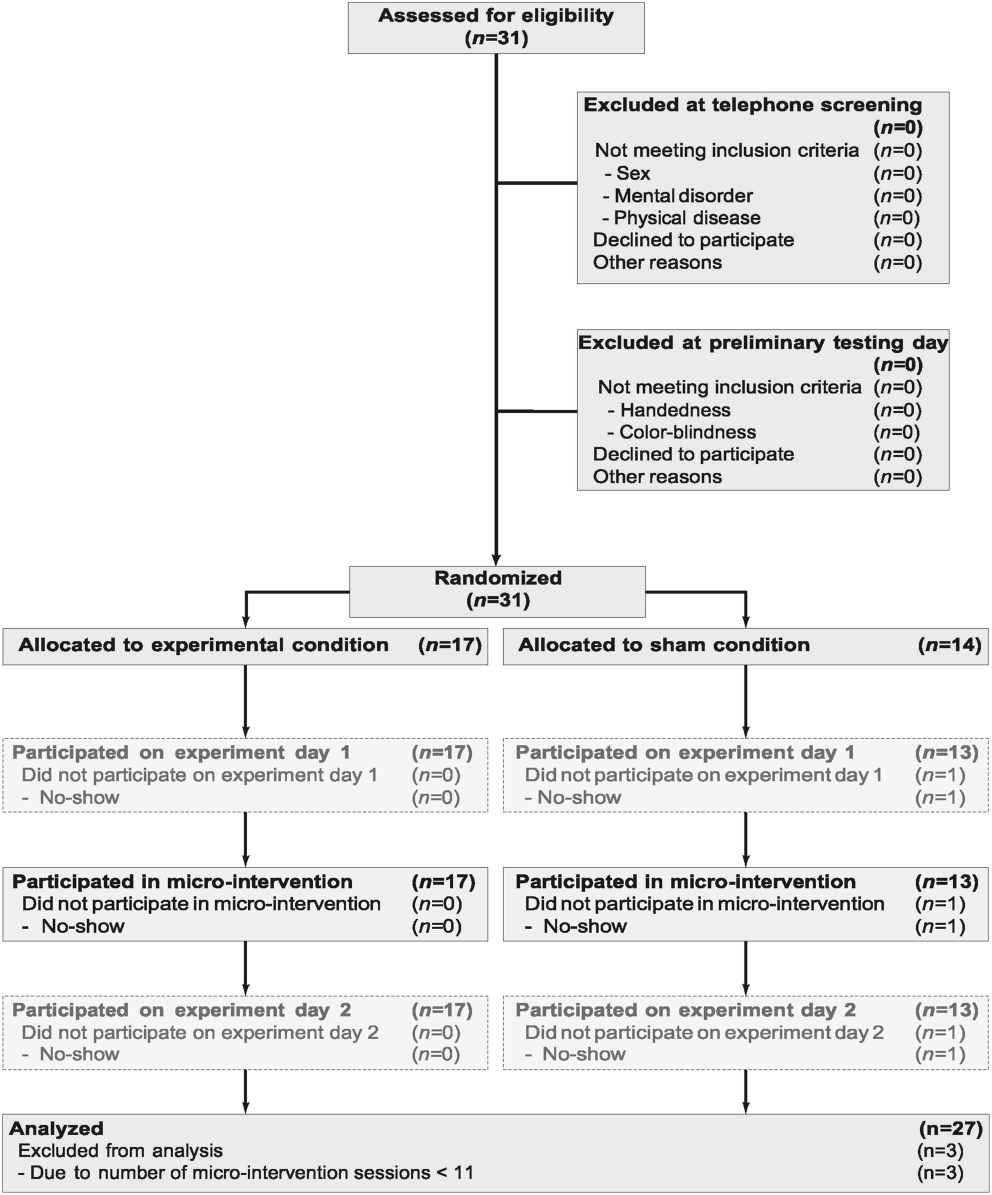
**Participant flow through study**.

The flowchart of participants is provided in Figure 2. From the 31 subjects included in the study, one participant did not show up on experiment day 1 and hence neither received instructions for nor participated in any smartphone-based micro-intervention. Three other subjects did participate in <3 micro-intervention sessions (one subject participated in 1 session and two subjects participated in 2 sessions) and were hence excluded from further analyses. All subjects were males of Korean nationality. Characteristics of the study sample on which the analyses are based (*N* = 27) are provided in Table 2. (For the sake of transparency, characteristics of the full study sample (*N* = 30) is provided in Supplementary Material Table 3).

### Descriptive information on smartphone-based micro-intervention sessions

**Figure 3:**
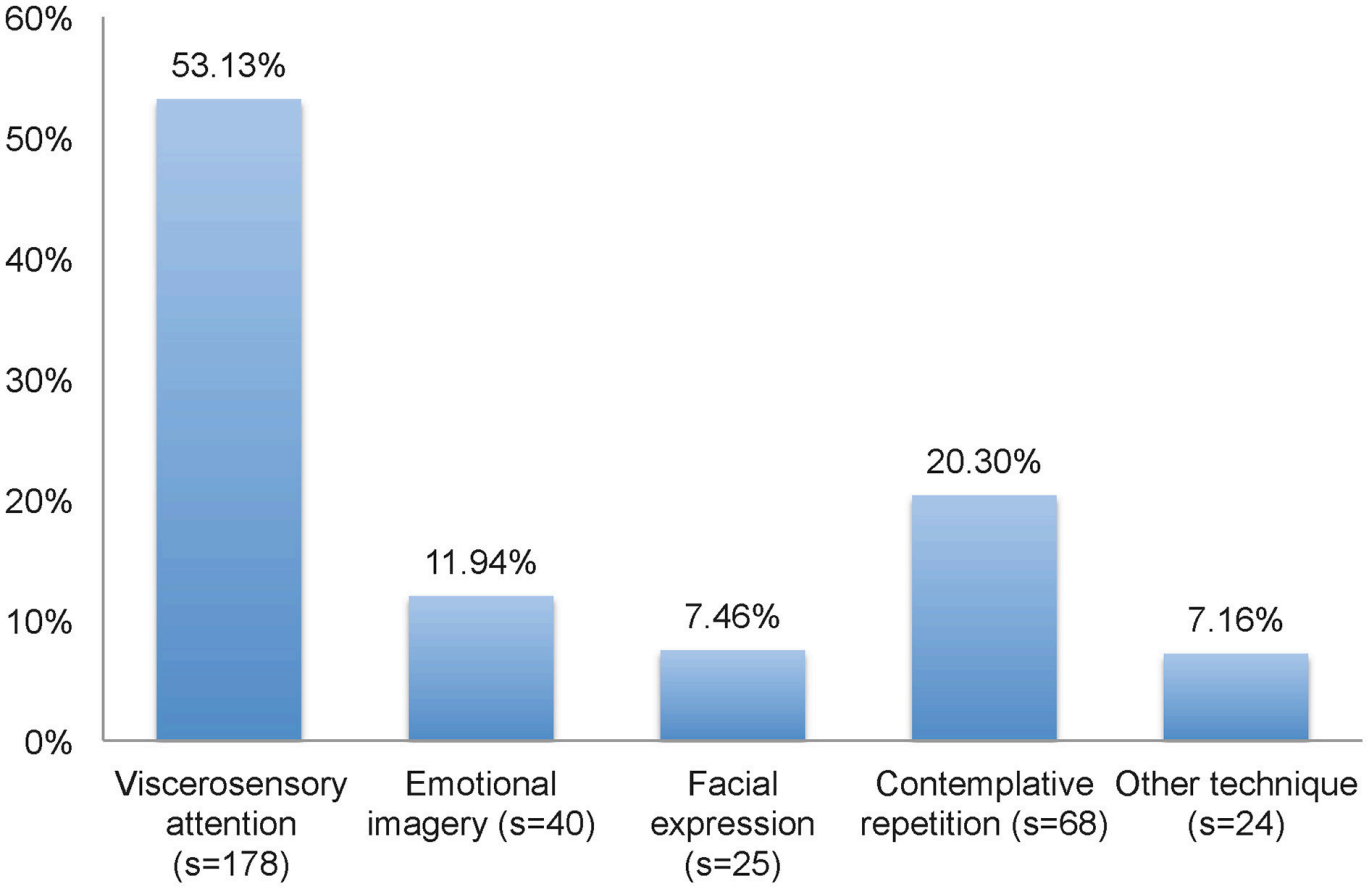
**Relative frequency of psychotherapeutic techniques applied during the smartphone-based micro-intervention sessions**. Information on selected technique missing for one session; s, number of sessions.

The 27 subjects participated in 336 out of 351 possible smartphone-based micro-intervention sessions in total (95.73%). The mean number of micro-intervention sessions per subject was 12.44 (standard deviation, *SD* = 0.80, Range: 11–13) [respective information regarding the sample of *N* = 30 participants, who in total participated in 342 out of 390 possible micro-intervention sessions (87.69%) is provided in Supplementary Material Table 3]. 26 sessions (7.74%) were conducted at 0800 h or later but before 0900 h, 63 sessions (18.75%) were conducted at 0900 h or later but before 1200 h, 54 sessions (16.07%) were conducted at 1200 h or later but before 1500 h, 44 sessions (13.10%) were conducted at 1500 h or later but before 1800 h, 69 sessions (20.54%) were conducted at 1800 h or later but before 2100 h, 69 sessions (20.54%) were conducted at 2100 h or later but before 0000 h, and 11 sessions (3.27%) were conducted at 0000 h or later but before 0300 h. The relative frequency of psychotherapeutic techniques applied during the micro-intervention sessions is depicted in Figure 3.

### Main results from the mixed model analyses

**Figure 4:**
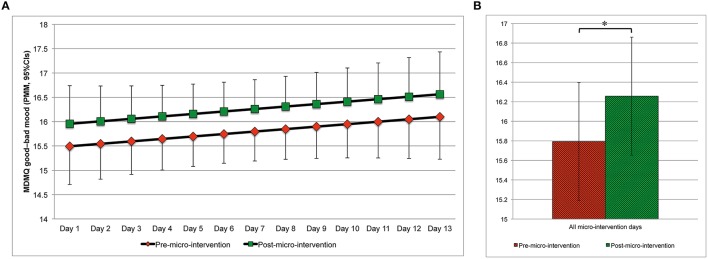
**Good–bad mood (predicted marginal means, 95%CIs) pre- and post-micro-intervention, by the micro-intervention day (A) and across all micro-intervention days (B)**. ^*^b = 0.464, 95%CI: 0.068–0.860, *t*_(613.3)_ = 2.298, *p* = 0.022; Higher values indicate better mood; In **(A)**, for the pre-micro-intervention values, the lower boundaries of the 95%CIs are depicted, for the post-micro-intervention values, the upper boundaries of the 95%CIs are depicted; CI, confidence interval; MDMQ, Multidimensional Mood State Questionnaire; PMM, predicted marginal means.

**Figure 5:**
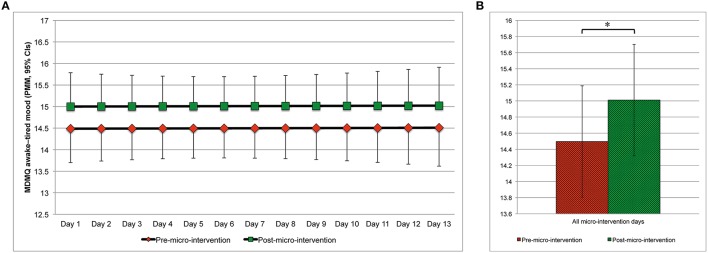
**Awake–tired mood (predicted marginal means, 95%CIs) pre- and post-micro-intervention, by the micro-intervention day (A) and across all micro-intervention days (B)**. ^*^b = 0.514, 95%CI: 0.103–0.924, *t*_(612.4)_ = 2.456, *p* = 0.014; Higher values indicate being more awake; In **(A)**, for the pre-micro-intervention values, the lower boundaries of the 95%CIs are depicted, for the post-micro-intervention values, the upper boundaries of the 95%CIs are depicted; CI, confidence interval; MDMQ, Multidimensional Mood State Questionnaire; PMM, predicted marginal means.

**Figure 6:**
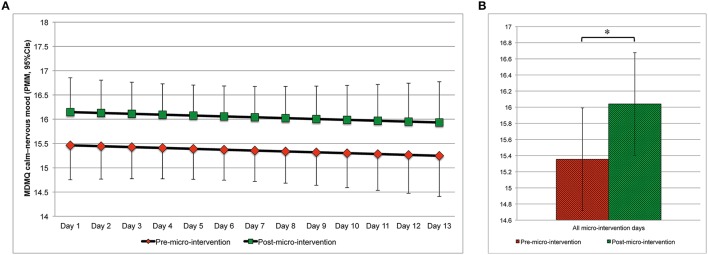
**Calm–nervous mood (predicted marginal means, 95%CIs) pre- and post-micro-intervention, by the micro-intervention day (A) and across all micro-intervention days (B)**. ^*^b = 0.685, 95%CI: 0.360–1.010, *t*_(612.3)_ = 4.137, *p* < 0.001; Higher values indicate being calmer; In **(A)**, for the pre-micro-intervention values, the lower boundaries of the 95%CIs are depicted, for the post-micro-intervention values, the upper boundaries of the 95%CIs are depicted; CI, confidence interval; MDMQ, Multidimensional Mood State Questionnaire; PMM, predicted marginal means.

**Figure 7:**
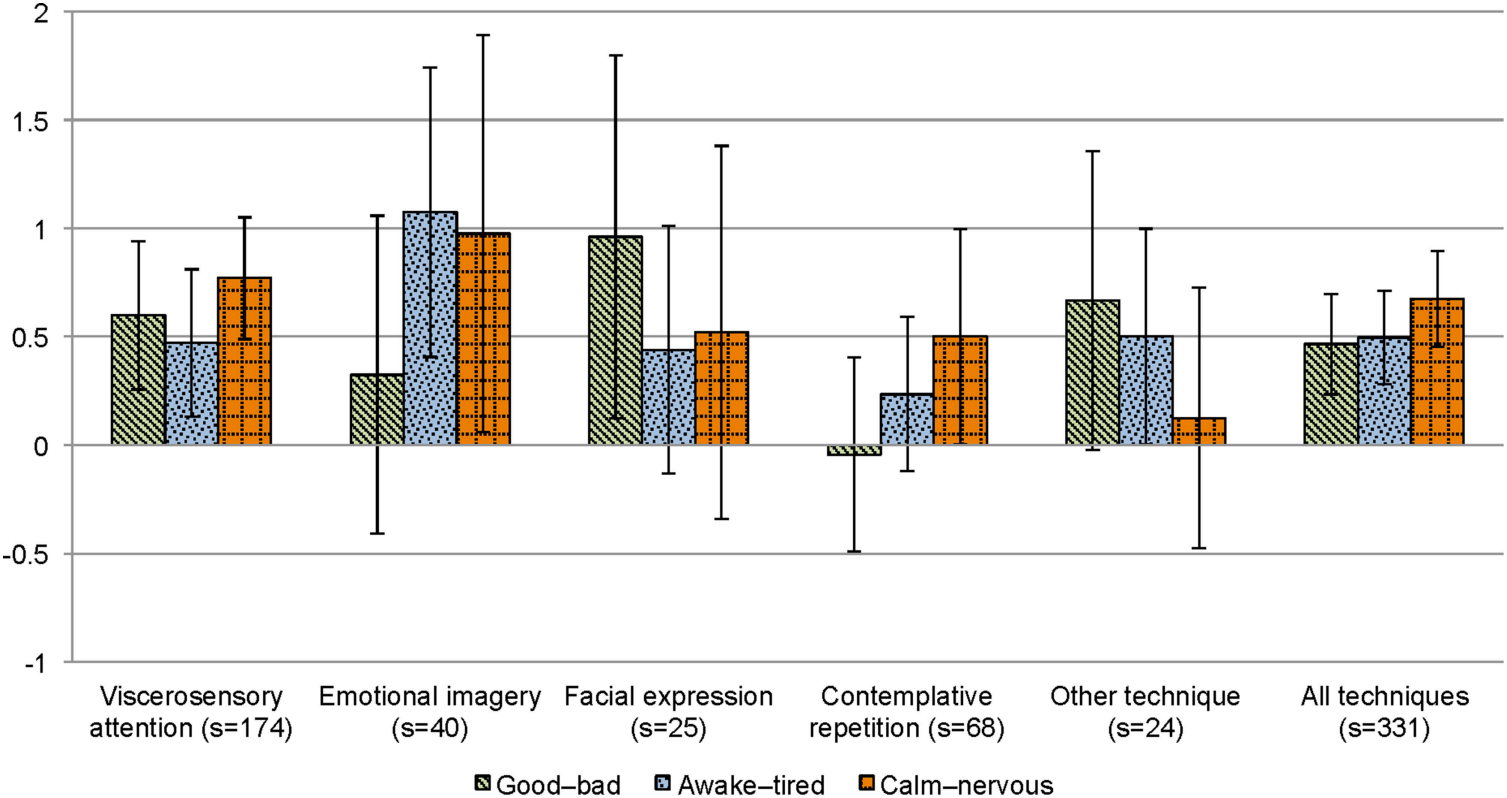
**Mood changes (means, 95%CIs) from pre- to post-micro-intervention, stratified according to psychotherapeutic technique**. Depending on the scale, higher values indicate better mood, being more awake, and calmer, respectively; CI, confidence interval; s, number of sessions.

Changes in mood from pre- to post-micro-intervention and across micro-intervention days are depicted in Figure 4 (MDMQ good-bad mood; Figures 4B,A, respectively), Figure 5 (MDMQ awake-tired mood; Figures 5B,A, respectively), and Figure 6 (MDMQ calm-nervous mood; Figures 6B,A, respectively). Mood changes stratified according to psychotherapeutic technique are depicted in Figure 7. All mixed models included a random intercept and slope of day varying among subjects. Entering “micro-intervention day” as higher order than linear polynomial did not improve model fit. In all three mixed models (with GB, AT, and CN as outcome) none of the interaction terms were statistically significant (see Supplementary Material Table 4 for related statistical parameters) and they were hence removed from the models. This means that there was no indication that changes in mood from pre- to post-micro-intervention differed across micro-intervention days, between psychotherapeutic techniques, and between conditions.

With regard to good or bad mood as outcome, mood improved from pre- to post-micro-intervention [*b* = 0.464, 95%CI [0.068, 0.860], *t*_(613.3)_ = 2.298, *p* = 0.022]. Increases in mood across days were statistically non-significant [*b* = 0.051, 95%CI [–0.039, 0.140], *t*_(26.8)_ = 1.112, *p* = 0.276]. With regard to awake–tired (AT) mood as outcome, subjects became more awake from pre- to post-micro-intervention [*b* = 0.514, 95%CI [0.103, 0.925], *t*_(612.4)_ = 2.456, *p* = 0.014], but not across days [*b* = 0.002, 95%CI [−0.073, 0.077], *t*_(25.5)_ = 0.048, *p* = 0.962]. With regard to calm–nervous (CN) mood as outcome, subjects became calmer from pre- to post-micro-intervention [*b* = 0.685, 95%CI [0.360, 1.010], *t*_(612.3)_ = 4.137, *p* < 0.001], but not across days [*b* = −0.018, 95%CI [−0.088, 0.052], *t*_(26.3)_ = 0.502, *p* = 0.620].

### Additional results regarding mood changes, and participants' feedback

Average mood improvements from pre- to post-micro-interventions across day 2 to day 12 were significantly associated with an increase in mood pre-micro-interventions from day 1 to day 13 with regard to GB mood (*r* = 0.614, 95%CI [0.297, 0.809], *p* < 0.001), but not AT mood (*r* = 0.279, 95%CI [−0.122, 0.602], *p* = 0.167) and CN mood (*r* = 0.277, 95%CI [−0.124, 0.601], *p* = 0.170) (calculations based on *n* = 26, due to missing data).

The feedback of the participants (*N* = 27) regarding the number of days of the smartphone-based micro-intervention revealed that 5 subjects (18.52%) agreed that 2 weeks were too short to be successful, while 10 subjects (37.04%) disagreed (the other 12 subjects were neutral); 13 subjects (48.15%) agreed that 2 weeks were well tolerable, while 3 subjects (11.11%) disagreed (the other 11 subjects were neutral). Regarding the duration of the sessions, 8 subjects (29.63%) agreed that the duration was too short to be successful, while 13 subjects (48.10%) disagreed (among which one subject even “strongly disagreed”; the other 6 subjects were neutral); 18 subjects (66.66%) agreed (among which one subject even “strongly agreed”) that the duration was well tolerable, while 2 subjects (7.40%) disagreed (among which one subject even “strongly disagreed”) (the other seven subjects were neutral). More detailed information on the feedback, as well as respective information based on the sample of participants who at least received the micro-intervention instructions (*N* = 30) with relative frequency of responses virtually identical to those reported here, are provided in Supplementary Material Table 2.

## Discussion

The aim of this study was to scrutinize in a real-world setting the use of smartphone-based micro-interventions in form of psychotherapeutic techniques and related changes in mood in a non-clinical sample. We hypothesized that mood improved from pre- to post micro-intervention sessions. Our hypothesis was confirmed. Subjects reported better mood and being calmer and more awake at post- as compared to pre-micro-intervention. However, there was no indication of increases in mood across days. Notably, greater mood improvements (GB mood) from pre- to post-micro-intervention were associated with overall changes in mood from the 1st day until the last day, which would be in line with micro-interventions incrementally improving mood across days if successful on individual days, even though our study design does not allow inferring causality or making assumptions about the long-term stability of the effects. There was no indication that mood improvements from pre- to post-micro-intervention differed between techniques or across the 13 micro-intervention days; hence there was no evidence for habituation of potential micro-intervention effects.

Participants conducted the vast majority of the requested micro-interventions sessions, and only a minority of subjects provided negative feedback regarding the number of micro-intervention days or the duration of the sessions. This indicates that a repeated application of smartphone-based micro-intervention sessions is generally well tolerated. Still, some individuals reported that they would have preferred a higher or lower number of sessions or a longer or shorter training duration, which indicates that personalization also of these parameters may have the potential to further improve the acceptance of smartphone-based micro-interventions.

Our findings extend previous evidence that short-term interventions, using different strategies, can modify mood in non-clinical samples in well-controlled laboratory settings (Velten, 1968; Martin, 1990; Schaefer et al., 2010), by indicating that this holds true when interventions are applied via smartphone in a real-world setting. They are in line with preliminary laboratory-based evidence that smartphone-based interventions can elicit positive mood states (e.g., Cipresso et al., 2012).

With regard to studies with clinical samples assessing psychotherapeutic face-to-face settings, our findings are in line with evidence that mindfulness-based strategies can improve mood and distress (e.g., Brake et al., 2016), even though the current study was only performed on a non-clinical sample of participants. A recent meta-analysis reported that online mindfulness-based intervention programs of 2–12 weeks duration were effective to reduce symptoms of mental disorders, notably with larger effect sizes for interventions of longer duration (Spijkerman et al., 2016). Furthermore, there is preliminary evidence that smartphone-based mindfulness intervention programs, lasting one to several weeks, may improve mood and reduce stress or symptoms of mental disorders (e.g., Brake et al., 2016). Our findings—notably based on a non-clinical sample of participants—are in line with this observation, indicating that (i) mindfulness-based micro-interventions of only several minutes duration, applied via smartphone, go along with rapid mood improvements, and (ii) if these interventions are successful during daily individual sessions, they are potentially leading to mood improvements across 2 weeks, even though we cannot make any assumptions about potential longer-term effects. Furthermore, our findings are in line with evidence from the field of smoking cessation, indicating that mobile phones (however, primarily via text messaging), have been successfully used to trigger behavior change (Whittaker et al., 2016). Notably, studies on smartphone-based interventions that target mental health related behavior do not always provide evidence that the interventions have the intended effects; in contrast, some interventions may even lead to opposite effects, at least in subgroups (Gajecki et al., 2014).

Our study design does not allow to disentangle potential processes underlying the mood changes observed in our study, and to identify to which extent different features of the intervention may have contributed to the observed mood changes and the overall good engagement of study participants with the digital intervention. One may speculate that the use of prompts and reminders in our study has improved digital engagement, in line with what has been previously shown (Alkhaldi et al., 2016). Further, consistent with previous evidence, the personal encounter between study personnel and the participants preceding the real-world micro-interventions may have enhanced intervention effects and digital engagement (Palmqvist et al., 2007; Spek et al., 2007; Andersson and Cuijpers, 2009; Richards and Richardson, 2012; Baumeister et al., 2014).

Important strengths of this study include, first, the use of psychotherapeutic techniques for which previous evidence indicated potential to improve stress-related processes; second, the participants' individual selection of their preferred techniques and, third, individual selection of training times; fourth, the use of video-clip supported procedures, ensuring a standardized application of the micro-intervention; and fifth, the use of mixed model analyses, taking into account, amongst others, individual mood variations across days.

There are also limitations. First, we did not include a randomized control condition. Therefore, we cannot determine which factors led to the mood related changes, and we cannot exclude changes in mood driven by digital placebo effects (Torous and Firth, 2016), which, however, is an issue not only in our study but in numerous other studies on the effects of psychotherapeutic interventions (Ioannidis, 2016). Future studies should estimate the effects of micro-interventions on mood within larger randomized controlled trials. Second, the data presented here were collected within a larger study. We cannot finally exclude that our findings were influenced by procedures during the preceding study days. However, there was no statistically significant association between the experimental condition participants were assigned to on experiment day 1 and smartphone-based micro-intervention-related mood changes during the 13 real-world sessions, making it rather unlikely that the randomization within the framework of the larger study was of substantial relevance. Third, the study sample was rather homogenous, with all participants being male and the majority being rather experienced using smartphones. Notably, given that males seek less traditional face-to-face treatment for mental health issues than females (Rhodes et al., 2002), males may be of special interest as target group for alternative interventive approaches. Our findings should be generalized with caution, and future studies are needed that target populations of different cultural backgrounds and more heterogeneous with regard to sex, age, educational background, and digital literacy. Fourth, even though participants were of Korean nationality, having Korean as a first language, written study material was provided in English, which was not adapted or normed to the local population. However, all participants had excellent knowledge of written English, and using the English versions of assessment instruments ensured that well-validated versions were applied. Fifth, without follow-up assessment, we cannot draw any conclusion regarding the long-term stability of the mood changes. Finally, we did not randomize the order in which the different techniques were introduced to the participants. Hence, we cannot exclude that order of introduction may have influenced the individual choice of techniques. However, identifying differences in mood changes across psychotherapeutic techniques was not the main goal of our study, and we would have needed a larger sample size and randomized assignment to techniques to further scrutinize this question. Notably, allowing participants to select the technique of their choice increased external validity of our study design, as smartphone-users usually substantially participate in the choice of apps that they apply, and the individual selection may also have improved engagement (Schueller, 2010).

Our findings may have different implications. They suggest the applicability of smartphone-based micro-interventions based on techniques that have been previously applied across a range of therapeutic approaches, including mindfulness-based psychotherapy. If our findings are corroborated in randomized controlled settings and different patient groups, targeted smartphone-based micro-interventions may represent a promising tool to modify mood in real-world settings, as part of more complex behavioral intervention technologies (BITs; Mohr et al., 2014), and complementing other psychotherapeutic interventions within blended treatments (Ly et al., 2015), and in line with the precision medicine approach (Insel, 2014; Collins and Varmus, 2015). Furthermore, they may be used to provide in-the-moment support for non-clinical populations to improve their mood, and allow delivering state-of-the-art psychotherapeutic techniques in a non-stigmatizing fashion to individuals who otherwise would not have access to therapy (Morris et al., 2010).

As mentioned above, future randomized controlled trials are needed to further scrutinize the effects of smartphone-based micro-interventions on mood, including studies addressing in detail the underlying mechanisms. Notably, alternative methodological frameworks, such as the “Continuous Evaluation of Evolving Behavioral Intervention Technologies (CEEBIT)” approach (Mohr et al., 2013b) or the “Person-Based Approach to Intervention Development” (Yardley et al., 2015), may allow evaluating the micro-interventions in different application contexts and help to further tailor applications based on the micro-interventions toward the target user population. In this context, we acknowledge that techniques used in the present study (i.e., the micro-interventions based on video-clips accessed via smartphones) may be considered rather “conventional,” in light of the rapid technological advancements made in smartphone technology. More recently, more advanced techniques, such as game-like applications, have been used for delivering smartphone-based psychotherapeutic interventions (e.g., Franklin et al., 2016). However, we also note that the advantage of our approach is that the video-clips can be easily integrated into more complex interventive “apps” (mobile phone applications) or within the contexts of communication routes, for example social media or messenger services (Dinakar et al., 2015). These approaches, with proper steps taken to safeguard information privacy, may confer low-barrier psychosocial interventions. With regard to technological advances, one may also consider combining the approach with the collection of information based on ambulatory biomarkers (Ben Khelil et al., 2011; Tegethoff et al., 2011; Choi et al., 2014), which may allow the application of micro-interventions based on multi-source information. Besides this, future studies should elucidate emerging questions, such as (i) to which extend the mood changes related to the micro-interventions depend on preceding personal contact with the subjects undergoing the micro-intervention, (ii) to which extend mood changes depend on whether subjects self-selected the type of technique applied, and (iii) whether individual mood changes triggered by a micro-intervention session can be predicted by contextual or time factors (Paredes et al., 2014), which will provide a basis for the further personalization of interventions.

Taken together, we provided evidence that smartphone-based micro-interventions are well-tolerated and go along with improvements in mood. In line with the precision medicine approach, smartphone-based micro-interventions may represent a promising tool to modify mood in real-world settings.

## Author contributions

GM, JL, SY, and MT designed the study; GM, JL, ES, and MT prepared the study material and data acquisition; JL, ES, MO, EJ, and HK recruited participants and acquired the data; ES, AB, and JA entered the data and prepared it for statistical analyses; GM, ES, and AB analyzed the data; GM, JL, and MT interpreted the data; GM wrote the first draft of the manuscript; GM, JL, ES, AB, MO, EJ, HK, JA, SY, and MT critically revised the manuscript for important intellectual content; MT obtained funding; All authors gave final approval of the manuscript version to be published and agreed to be accountable for all aspects of the work in ensuring that questions related to the accuracy or integrity of any part of the work are appropriately investigated and resolved.

## Funding

GM, JL, and MT receive funding from the National Research Foundation of Korea (NRF) within the Global Research Network Program (under project no. 2013S1A2A2035364). MT receives funding from the Swiss National Science Foundation, SNSF (project no. PZ00P1_137023). JL receives funding from the NRF, Ministry of Science, ICT, and Future Planning, Korea (2015R1A2A2A03004462) and the Korean Health Technology R&D Project, Ministry of Health and Welfare, Korea (HI12C1847); GM receives funding from the SNSF (project no. 100014_135328).

### Conflict of interest statement

GM acts as consultant for Janssen Research & Development, LLC. The other authors declare that the research was conducted in the absence of any commercial or financial relationships that could be construed as a potential conflict of interest.
