# Supplementary material for: Smartphone-Based Psychotherapeutic Micro-Interventions to Improve Mood in a Real-World Setting
Source: Front Psychol. 2016 Jul 28;7:1112. doi: 10.3389/fpsyg.2016.01112 (PMC4963605; doi:10.3389/fpsyg.2016.01112)
Supplement: Supplementary file 1 [file DataSheet1.PDF]

Supplementary Material

Smartphone-based psychotherapeutic micro-interventions to improve mood in a real-world setting

Gunther Meinlschmidt, Jong-Hwan Lee, Esther Stalujanis, Angelo Belardi, Minkyung Oh, Eun Kyung Jung, Hyun-Chul Kim, Janine Alfano, Seung-Schik Yoo, Marion Tegethoff*

***Correspondence:** Marion Tegethoff: marion.tegethoff@unibas.ch

Supplementary Material Data Sheet 1. Brief outline of the larger study from which the data were derived

Study procedure of experiment day 1 and 2 (further details available from the authors on request): Subjects underwent a real-time functional magnetic resonance imaging neurofeedback (RT-fMRI NF) procedure, during which they repeatedly applied previously learned psychotherapeutic techniques inside an magnetic resonance imaging (MRI) scanner. Furthermore, the experiment included a Stroop task, resting periods, repeated blood pressure measurements, ratings on the Self-Assessment Manikin (SAM) scales, and saliva collections. For the RT-fMRI NF experiment only, subjects were randomly assigned to two conditions (details on the randomization procedure available from the authors on request), either the experimental or control condition, with subjects in the experimental condition (n=14 of those included in the main analyses of this article) receiving feedback derived from their own brain activity (blood oxygen level dependent (BOLD) signal) and subjects in the control condition (n=13 of those included in the main analyses of this article) receiving sham feedback (with the feedback signal derived from another subject's brain activity). Subjects were blinded regarding the condition they were assigned to. Neither the RT-fMRI NF procedure nor the randomization to the experimental or control condition is part of the research question of the present publication. Subjects' assignments to experimental or control condition was entered as covariate in some of our statistical analyses (see method section). After the RT-fMRI NF session on experiment day 1, we instructed subjects in how to perform the smartphone-based micro-interventions.
