# Supplementary material for: Smartphone-Based Psychotherapeutic Micro-Interventions to Improve Mood in a Real-World Setting
Source: Front Psychol. 2016 Jul 28;7:1112. doi: 10.3389/fpsyg.2016.01112 (PMC4963605; doi:10.3389/fpsyg.2016.01112)
Supplement: Supplementary file 2 [file DataSheet2.PDF]

Supplementary Material

Smartphone-based psychotherapeutic micro-interventions to improve mood in a real-world setting

Gunther Meinlschmidt, Jong-Hwan Lee, Esther Stalujanis, Angelo Belardi, Minkyung Oh, Eun Kyung Jung, Hyun-Chul Kim, Janine Alfano, Seung-Schik Yoo, Marion Tegethoff*

***Correspondence:** Marion Tegethoff: marion.tegethoff@unibas.ch

Supplementary Material Data Sheet 2. Structure and transcript of content of the micro-interventions provided within video-clips (see Supplementary Material Video 1 to 5 for the video-clips themselves)

The total duration of each video-clip is approximately 4 minutes and 40 seconds. The structure of the video-clips is comparable across all psychotherapeutic techniques and contains 4 phases:

1) Each video-clip starts with the display of an instruction text (duration of display approximately 10 seconds): “During the training, you may notice that it is not easy to maintain concentration. Whenever you realize that your thoughts are running away, please just get back to the task and try again. Good luck with the training!”
2) This is followed by a phase in which subjects are asked to apply the psychotherapeutic technique with an instruction text displayed, depending on the technique (duration of phase approximately 120 seconds):
    a. Technique ‘viscerosensory attention’; Text displayed: “Your task: Shift your attention towards versus away from bodily sensations, for example your heartbeat, breathing, or feelings in stomach. Keep your attention focused on each sensation for a while.”
    b. Technique ‘emotional imagery’; Text displayed: “Your task: Imagine emotionally positive, negative or neutral situations, and shift your attention between them, for example think of a beloved person, a stressful exam or a conflict, or a bus ride… Keep your attention focused on each situation for a while.”
    c. Technique ‘facial expression’; Text displayed: “Your task: Make different emotional expressions with your face and keep each for a while (e.g. happy face, angry face, neutral face).”
    d. Technique ‘contemplative repetition’; Text displayed: “Your task: Repeat a short, very easy sentence, or slowly count from 1 to 10 (repeat this over and over again).”
    e. Technique ‘other’; Text displayed: “Your task: Remember the strategy that you have successfully practiced in the scanner. Please concentrate and practice this strategy during the next minutes.”

   ➔ During this phase, the time until the end of this phase is shown at the bottom right side of the screen and changes every 30 seconds, starting with the display “2 minutes to go”, followed by “90 seconds to go”, followed by “60 seconds to go”, and followed by “30 seconds to go”. An hourglass icon at the left of the displayed text illustrates that the text refers to the remaining time.

The end of this phase is indicated by the text: “round 1 finished -- thank you --” (duration of display approximately 4 seconds).

3) This is followed by a short break during which another text is displayed (duration of display approximately 30 seconds, text is scrolling from bottom to top): “Pause: 30 seconds… You have done a great job! You may have noticed that it is not easy to maintain concentration during such a challenging task. Whenever you realize that your thoughts are running away, please just get back to the task and try again. Good luck for the next round!”.
4) This is followed by a phase identical to phase 2 outlined above, with the exception of the end of the phase being indicated by the text: “Congratulations, you have finished today’s training!” (duration of display approximately 4 seconds).

The beginning and end of phases 2 and 4 are signalized by the sound of two high-pitched gongs at approximately 1.7 seconds interval. During phases 2 and 4, images illustrating the respective techniques are shown as background images (see information provided with Supplementary Material 2 to 6 above), while the instruction text is displayed in black font with white outline for the techniques ‘viscerosensory attention’ and ‘other’, and technique instruction text displayed in white font with black outline for the techniques ‘emotional imagery’, ‘facial expression’, and ‘contemplative repetition’, and text to indicate the end of the phases displayed in white font with black outline. During phases 1 and 3, the background screen is dark blue, with text displayed in white font.
