# Supplementary material for: Smartphone-Based Psychotherapeutic Micro-Interventions to Improve Mood in a Real-World Setting
Source: Front Psychol. 2016 Jul 28;7:1112. doi: 10.3389/fpsyg.2016.01112 (PMC4963605; doi:10.3389/fpsyg.2016.01112)
Supplement: Supplementary file 3 [file DataSheet3.PDF]

Supplementary Material

Smartphone-based psychotherapeutic micro-interventions to improve mood in a real-world setting

Gunther Meinlschmidt, Jong-Hwan Lee, Esther Stalujanis, Angelo Belardi, Minkyung Oh, Eun Kyung Jung, Hyun-Chul Kim, Janine Alfano, Seung-Schik Yoo, Marion Tegethoff*

***Correspondence:** Marion Tegethoff: marion.tegethoff@unibas.ch

Supplementary Material Data Sheet 3. Additional information regarding the videos provided as 'Supplementary Material Video 1 to 5'

Video to guide through the micro-intervention 'Viscerosensory attention': Video-format: Video encoded via Moving Picture Experts Group (MPEG)-4 Part 10, Advanced Video Coding (MPEG-4 AVC; "H.264"); duration: approximately 4 minutes and 40 seconds); Please note: The background picture displayed during this video is a modified version of *Male with organs* by *Mikael Häggström*, available via Wikimedia commons under the Creative Commons CC0 1.0 Universal Public Domain Dedication; retrieved from: https://commons.wikimedia.org/wiki/File:Male_with_organs.png

Video to guide through the micro-intervention 'Emotional imagery': Video-format: Video encoded via MPEG-4 AVC; duration: approximately 4 minutes and 40 seconds; Please note: The background picture displayed during this video is a modified version of *Meditation in Wat Khung Taphao* by *Tevaprapas*, available via Wikimedia commons under the Creative Commons Attribution 2.5 Generic license; retrieved from: https://commons.wikimedia.org/wiki/File:Meditation_in_Wat_Khung_Taphao.jpg

Video to guide through the micro-intervention 'Facial expression': Video-format: Video encoded via MPEG-4 AVC; duration: approximately 4 minutes and 40 seconds; Please note: The background picture displayed during this video is a modified version of *Haitian Grill (8131305206)* by *Alex Proimos*, available via Wikimedia Commons under the Creative Commons Attribution 2.0 Generic license; retrieved from: https://commons.wikimedia.org/wiki/File:Haitian_Grill_(8131305206).jpg

Video to guide through the micro-intervention 'Contemplative repetition': Video-format: Video encoded via MPEG-4 AVC; duration: approximately 4 minutes and 40 seconds; Please note: The background picture displayed during this video is a modified version of *Mantras caved into rock in Tibet* by *Nathan Freitas*, available via Wikimedia Commons under the Creative Commons Attribution-Share Alike 2.0 Generic license; retrieved from: https://commons.wikimedia.org/wiki/File:Mantras_caved_into_rock_in_Tibet.jpg

Video to guide through the micro-intervention 'Other technique': Video-format: Video encoded via MPEG-4 AVC; duration: approximately 4 minutes and 40 seconds; Please note: The background picture displayed during this video is a modified version of *Nicolas P. Rougier's rendering of the human brain* by *Nicolas Rougier*, available via Wikimedia Commons under the GNU General Public License; retrieved from: https://commons.wikimedia.org/wiki/File:Nicolas_P._Rougier%27s_rendering_of_the_human_brain.png?uselang=de
