# Supplementary material for: Smartphone-Based Psychotherapeutic Micro-Interventions to Improve Mood in a Real-World Setting
Source: Front Psychol. 2016 Jul 28;7:1112. doi: 10.3389/fpsyg.2016.01112 (PMC4963605; doi:10.3389/fpsyg.2016.01112)
Supplement: Supplementary file 4 [file Table1.PDF]

Supplementary Material

Smartphone-based psychotherapeutic micro-interventions to improve mood in a real-world setting

Gunther Meinlschmidt, Jong-Hwan Lee, Esther Stalujanis, Angelo Belardi, Minkyung Oh, Eun Kyung Jung, Hyun-Chul Kim, Janine Alfano, Seung-Schik Yoo, Marion Tegethoff*

***Correspondence:** Marion Tegethoff: marion.tegethoff@unibas.ch

Supplementary Material Table 1. Information on mobile devices, operating systems (OS), and Internet browsers used for micro-intervention participation[1] (*N*=27).

Variable	Category	*n*	(%)[2]
Smartphone type			
	Samsung[3]	15	(55.56%)
	iPhone[4]	6	(22.22%)
	LG[5]	3	(11.11%)
	Nexus 7	1	(3.70%)
	SKY[6]	2	(7.40%)
OS			
	Android[7]	21	(77.78%)
	iOS[8]	6	(22.22%)
Internet browser			
	Chrome[9]	19	(70.37%)
	Android browser	5	(18.52%)
	Safari 6	3	(11.11%)

[1] *In case subjects used different smartphone types, OS, or Internet browsers across micro-intervention session days, we report information on the most common used combination of the three characteristics.*

[2] *Percentages may not total 100 due to rounding*

[3] *The following models were used: Samsung Galaxy S2 (SHW-M2505, SHW-M250K, SHV-E110S), Samsung Galaxy S3 (SHV-E210K, SHV-E210S, SHV-210L), Samsung Galaxy Note (SHV-E160K, SHV-E160L), and Samsung Galaxy Note 2 (SHV-E250K, SHV-E250L)*

[4] *No detailed information regarding iPhone models available*

[5] *The following models were used: LG-F220K Optimus GK and LG-F160K Optimus*

[6] *The following models were used: SKY Vega (IM-A8105) and SKY Vega Racer (IM-A770K)*

[7] *The following OS versions were used: Android 2.3.6, Android 4.0.4, Android 4.1.1, and Android 4.1.2*

[8] *The following OS versions were used: iOS 6 and iOS 7*

[9] *The following browser versions were used: Chrome 18, Chrome 28, Chrome 29, and Chrome 30*
