# Supplementary material for: Smartphone-Based Psychotherapeutic Micro-Interventions to Improve Mood in a Real-World Setting
Source: Front Psychol. 2016 Jul 28;7:1112. doi: 10.3389/fpsyg.2016.01112 (PMC4963605; doi:10.3389/fpsyg.2016.01112)
Supplement: Supplementary file 5 [file Table2.PDF]

Supplementary Material

Smartphone-based psychotherapeutic micro-interventions to improve mood in a real-world setting

Gunther Meinlschmidt, Jong-Hwan Lee, Esther Stalujanis, Angelo Belardi, Minkyung Oh, Eun Kyung Jung, Hyun-Chul Kim, Janine Alfano, Seung-Schik Yoo, Marion Tegethoff*

***Correspondence:** Marion Tegethoff: marion.tegethoff@unibas.ch

Supplementary Material Table 2. Participants feedback regarding the micro-intervention, based on the main sample, consisting of participants that conducted at least 3 micro-intervention sessions (*N*=27), and based on the sample of participants that received at least the micro-intervention instructions (*N*=30).

		N=27		*N*=30	
**Variable**	**Category**	***n***	**(%)***	***n***	**(%)***
"Two weeks of ambulatory training were too short to be successful."	Strongly agree	0	(0%)	0	(0%)
	Agree	5	(18.52%)	5	(16.67%)
	Neutral	12	(44.44%)	15	(50.00%)
	Disagree	10	(37.04%)	10	(33.33%)
	Strongly disagree	0	(0%)	0	(0%)
"Two weeks of ambulatory training were well tolerable."	Strongly agree	0	(0%)	0	(0%)
	Agree	13	(48.15%)	13	(43.33%)
	Neutral	11	(40.74%)	12	(40.00%)
	Disagree	3	(11.11%)	5	(16.67%)
	Strongly disagree	0	(0%)	0	(0%)
"Approximately 10 minutes of ambulatory training per day were too short to be successful."	Strongly agree	0	(0%)	0	(0%)
	Agree	8	(29.63%)	8	(26.67%)
	Neutral	6	(22.22%)	7	(23.33%)
	Disagree	12	(44.44%)	14	(46.67%)
	Strongly disagree	1	(3.70%)	1	(3.33%)
"Approximately 10 minutes of ambulatory training per day were well tolerable."	Strongly agree	1	(3.70%)	1	(3.33%)
	Agree	17	(62.96%)	17	(56.67%)
	Neutral	7	(25.93%)	9	(30.00%)
	Disagree	1	(3.70%)	2	(6.67%)
	Strongly disagree	1	(3.70%)	1	(3.33%)

**Percentages may not total 100 due to rounding.*
