# Supplementary material for: Smartphone-Based Psychotherapeutic Micro-Interventions to Improve Mood in a Real-World Setting
Source: Front Psychol. 2016 Jul 28;7:1112. doi: 10.3389/fpsyg.2016.01112 (PMC4963605; doi:10.3389/fpsyg.2016.01112)
Supplement: Supplementary file 6 [file Table3.PDF]

Supplementary Material

Smartphone-based psychotherapeutic micro-interventions to improve mood in a real-world setting

Gunther Meinlschmidt, Jong-Hwan Lee, Esther Stalujanis, Angelo Belardi, Minkyung Oh, Eun Kyung Jung, Hyun-Chul Kim, Janine Alfano, Seung-Schik Yoo, Marion Tegethoff*[*]

***Correspondence:** Marion Tegethoff: marion.tegethoff@unibas.ch

Supplementary Material Table 3. Characteristics of the sample of all subjects receiving micro-intervention instructions (*N*=30).

Categorical variables

Variable	Category	*n*	(%)*
Marital status			
	Single	22	(73.33%)
	In a relationship	8	(26.67%)
Highest degree			
	High school or equivalent	27	(90%)
	Bachelor's degree	3	(10%)
Size of household (including participant**)			
	1	2	(6.90%)
	2	0	(0%)
	3	1	(3.45%)
	4	23	(79.31%)
	5	3	(10.34%)
"I am very experienced in using smartphones"			
	Strongly agree	8	(26.67%)
	Agree	16	(53.33%)
	Neutral	4	(13.33%)
	Disagree	1	(3.33%)
	Strongly disagree	1	(3.33%)

Continuous variables

Variable (unit)	Mean	(*SD*)	Range [min, max]
Age (years)	24.28	(2.27)	[19.75, 28.70]
Full time education (years)	15.1	(1.37)	[12, 18]
Training participation (days)	11.4	(3.38)	[1, 13]

**Percentages may not total 100 due to rounding; **Information from one subject missing*
Abbreviations: max, maximum; min, minimum; SD, standard deviation.
