# Supplementary material for: Smartphone-Based Psychotherapeutic Micro-Interventions to Improve Mood in a Real-World Setting
Source: Front Psychol. 2016 Jul 28;7:1112. doi: 10.3389/fpsyg.2016.01112 (PMC4963605; doi:10.3389/fpsyg.2016.01112)
Supplement: Supplementary file 7 [file Table4.PDF]

Supplementary Material

Smartphone-based psychotherapeutic micro-interventions to improve mood in a real-world setting

Gunther Meinlschmidt, Jong-Hwan Lee, Esther Stalujanis, Angelo Belardi, Minkyung Oh, Eun Kyung Jung, Hyun-Chul Kim, Janine Alfano, Seung-Schik Yoo, Marion Tegethoff*

***Correspondence:** Marion Tegethoff: marion.tegethoff@unibas.ch

Supplementary Material Table 4. Additional results in form of interaction effects of mixed model analyses predicting mood (*Number of observations*=667).

**Good–bad mood**						
**Parameter**	***beta***	***SE***	**95%CI [LB, UB]**	***t***	***df***	***p*-value**
pre- to post-micro-intervention × micro-intervention day	-0.056	0.054	[-0.162, 0.050]	-1.042	613.6	0.298
pre- to post-micro-intervention × technique: contemplative repetition vs. viscerosensory attention	-0.632	0.537	[-1.685, 0.421]	-1.176	613.1	0.240
pre- to post-micro-intervention × technique: other vs. viscerosensory attention	-0.144	0.468	[-1.062, 0.773]	-0.309	613.3	0.758
pre- to post-micro-intervention × condition	-0.105	0.404	[-0.898, 0.688]	-0.259	613.3	0.796
**Awake–tired mood**						
**Parameter**	***beta***	***SE***	**95%CI [LB, UB]**	***t***	***df***	***p*-value**
pre- to post-micro-intervention × micro-intervention day	-0.036	0.056	[-0.146, 0.074]	-0.644	612.7	0.520
pre- to post-micro-intervention × technique: contemplative repetition vs. viscerosensory attention	-0.248	0.558	[-1.342, 0.846]	-0.444	612.1	0.657
pre- to post-micro-intervention × technique: other vs. viscerosensory attention	0.094	0.486	[-0.859, 1.046]	0.193	612.4	0.847
pre- to post-micro-intervention × condition	-0.097	0.420	[-0.921, 0.726]	-0.231	612.3	0.817
**Calm–nervous mood**						
**Parameter**	***beta***	***SE***	**95%CI [LB, UB]**	***t***	***df***	***p*-value**
pre- to post-micro-intervention × micro-intervention day	-0.024	0.044	[-0.111, 0.064]	-0.535	612.6	0.593
pre- to post-micro-intervention × technique: contemplative repetition vs. viscerosensory attention	-0.236	0.441	[-1.100, 0.629]	-0.534	612.2	0.593
pre- to post-micro-intervention × technique: other vs. viscerosensory attention	-0.190	0.384	[-0.943, 0.563]	-0.494	612.3	0.621
pre- to post-micro-intervention × condition	-0.297	0.332	[-0.948, 0.355]	-0.893	612.3	0.372

Notes: CI, confidence interval; LB, lower bound; SE, standard error; UB, upper bound.
